# Role of the Ear in Meningitis: A Narrative Review of Neuroimaging

**DOI:** 10.3390/diagnostics15212733

**Published:** 2025-10-28

**Authors:** Teresa Perillo, Raffaella Capasso, Teresa Califano, Federica Cataldo, Ludovica Fulgione, Alessandra Scaravilli, Giuseppe Vitale, Antonio Pinto

**Affiliations:** Radiology Department, CTO Hospital, AORN dei Colli, 800131 Naples, Italy; dott.ssacapasso@gmail.com (R.C.);

**Keywords:** Meningitis, otology, central nervous system, magnetic resonance imaging, otomastoiditis, cholesteatoma, congenital ear malformation, neurology

## Abstract

**Background/Objectives**: Meningitis is a life-threatening condition often associated with ear pathology. This narrative review explores the critical role of the ear in Meningitis, emphasizing neuroimaging findings. **Methods**: Congenital malformations of the inner ear may significantly increase the risk of recurrent Meningitis, especially in children. Otomastoiditis, through direct extension or bony erosion, is a frequent cause of otogenic Meningitis. Cholesteatoma can erode bone and lead to severe intracranial complications, including Meningitis. Imaging is essential for early detection of bone defects, intracranial extension, and associated complications. Labyrinthitis and vestibulocochlear neuritis are usually complications of Meningitis. Less frequently, Meningitis may follow ear surgeries, such as cochlear implantation or mastoidectomy. **Conclusions**: Neuroradiologists must be aware of ear conditions associated with Meningitis to enable prompt diagnosis and correct management.

## 1. Introduction

Meningitis is defined as the inflammation of the meninges, which are connective tissue membranes that envelop the brain and spinal cord [1]. Meninges are divided into three layers, namely dura, arachnoid, and pia mater. Together, the arachnoid and pia mater are called the leptomeninges. The dura mater is the most superficial layer, whereas the leptomeninges are separated by the subarachnoid space, with the pia mater being the deepest layer and adhering to the brain [2].

Meningitis can be infectious or non-infectious in origin. Infective Meningitis is a severe, life-threatening condition that often leads to long-term consequences [3]. Common symptoms and clinical signs are fever, neck stiffness, confusion, or altered mental status, headache, sensitivity to light, nausea, and vomiting. Less frequently, clinical presentations include seizures, coma, and neurological deficits [4]. To diagnose Meningitis, a lumbar puncture is indicated to examine the cerebrospinal fluid (CSF), allowing the identification of the causative agent.

Imaging plays an important role, but findings are not specific in relation to the causative pathogen [1]. Computed tomography (CT) should be performed in patients with impairment of consciousness and/or neurologic deficits before lumbar puncture to exclude intracranial hypertension and/or brain herniations [2]. On the other side, magnetic resonance imaging (MRI) with intravenous contrast medium administration is the imaging modality of choice to detect abnormal meningeal enhancement, which is present in almost 50% of patients, and it is best seen on fluid-attenuated inversion recovery (FLAIR) weighted images than on post-contrast T1 sequences [5]. MRI is also useful to exclude mimics and evaluate the presence of complications, such as subdural empyema, epidural empyema, intraparenchymal brain abscesses, sigmoid or transverse sinus thrombosis, brain infarcts, petrous apicitis, and hydrocephalus [2].

**Figure 1:**
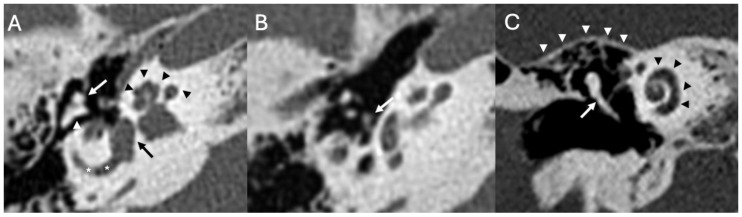
Axial (**A**,**B**) and coronal reformat CT (**C**) showing the ear ossicles, namely the malleus (white arrows in (**A**,**C**)), incus (white arrowhead in (**A**)), and the stapes (white arrow in (**B**)). The image also depicts the cochlea (black arrowheads in (**A**,**C**)), the vestibule (black arrow in (**A**)), the lateral semicircular canal (white asterisks in (**A**)) and the tegmen tympani (white arrowheads in (**C**)).

The temporal bone contains the structures of the external, middle, and inner ear [6]. The middle ear is an air-filled cavity containing the ossicular chain (malleus, incus, and stapes) (Figure 1) [7]. It has six walls. The roof is the tegmen tympani, a thin bony plate separating the tympanic cavity from the middle cranial fossa. The floor is the jugular wall, separating the tympanic cavity from the internal jugular vein. The lateral wall is formed by the tympanic membrane and epitympanic recess. The medial wall separates the middle from the inner ear, and it is composed of the promontory (which corresponds to the basal turn of the cochlea) and the round and oval windows. Posteriorly, there is the aditus ad antrum, representing the communication between the middle ear and the mastoid. The anterior wall separates the tympanic cavity from the carotid canal and inferiorly comprises the opening of the Eustachian tube. The inner ear includes a bony and a membranous component, together forming the bony and membranous labyrinth. The bony component comprises the cochlea, vestibule, superior, posterior, and lateral semicircular canals, and the vestibular aqueduct. The cochlea has a spiral form, with two-and-a-half and two-and-three-quarter turns around the modiolus. The vestibule is composed of the utricle, saccule, and basal end of the cochlear duct.

**Figure 2:**
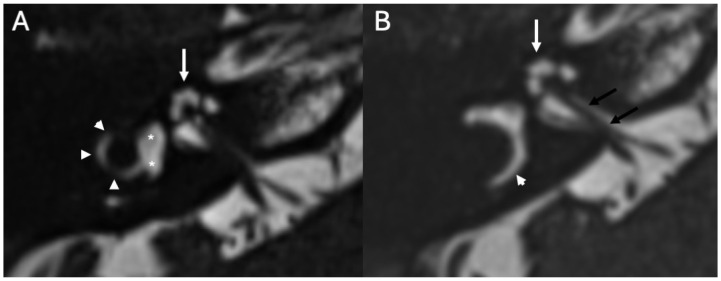
Axial heavily T2 weighted images (**A**,**B**) showing the cochlea (white arrows in (**A**,**B**)), the vestibule (asterisks in (**A**)), the lateral semicircular canal (arrowheads in (**A**,**B**)) and the vestibulo–cochlear nerve (black arrows in (**B**)).

CT is used to evaluate the bony structures and the ossicular chain. The acquisition protocol requires a slice thickness of 0.6 mm and a pixel resolution of 0.5 × 0.5 mm, with an appropriate window setting (window width 3500–4500 Hounsfield units and level 400–600 Hounsfield units) [7]. Given the complexity of the examined structures, multiplanar reconstructions are essential [8]. MRI is useful for visualizing the fluid-containing components of the inner ear, especially using heavily T2-weighted sequences [9] (Figure 2).

MRI sequence quality may be affected by the air in the ear cavity. To reduce artifacts, it is preferable to use turbo spin echo and spin echo sequences, high bandwidth, automatic or localized shimming, and smaller fields of view. CT is preferred to evaluate bony structures; therefore, it is useful in malformation, trauma, cholesteatoma, and chronic otomastoiditis. On the other hand, MRI enables evaluation of soft tissue and nerves; therefore, is the image modality of choice in labyrinthitis. MRI and CT are frequently used together for a complete evaluation, especially in malformation, cholesteatoma, and chronic otomastoiditis.

The aim of this narrative review is to describe the role of the ear in Meningitis, with a special focus on neuroimaging findings. A comprehensive literature search was conducted to identify peer-reviewed publications addressing otogenic and otology-related causes and complications of Meningitis, with particular attention to neuroimaging findings. The search encompassed studies indexed in PubMed/MEDLINE, Scopus, and Web of Science up to June 2025, using a combination of controlled vocabulary and free-text terms including “otogenic Meningitis,” “ear and Meningitis,” “temporal bone,” “otomastoiditis,” “cholesteatoma,” “inner ear malformation,” “labyrinthitis,” “cochlear implant Meningitis,” and “neuroimaging.” Boolean operators and truncation were applied to optimize search sensitivity and specificity. The principal studies are reported in Table 1.

## 2. Congenital Malformations

Congenital malformations of the petrous portion of the temporal bone may cause recurrent Meningitis [10]. In case of Meningitis associated with sensorineural hearing loss and CSF rhinorrhea or otorrhea, congenital anomalies of the temporal bone should be suspected. In this setting, the pathogenetic mechanism predisposing to recurrent Meningitis is related to the presence of a fistula between the middle ear cavity and the intracranial subarachnoid space, which may determine the leakage of CSF into the middle ear with the spread of infections in the intracranial compartment [11]. Congenital malformations are reported in 93% of children and 38% of adults with Meningitis [12]. Fistulas can be classified as perilabyrinthine or translabyrinthine [43]. Translabyrinthine fistulas are associated with an abnormal development of the cochlea, particularly with anomalies of the modiolus and the interscalar septum. On the other hand, perilabyrinthine fistulas may occur in cases of leakage of CSF through the oval window through a dehiscence in the basal plate of the stapes [10,44,45].

**Figure 3:**
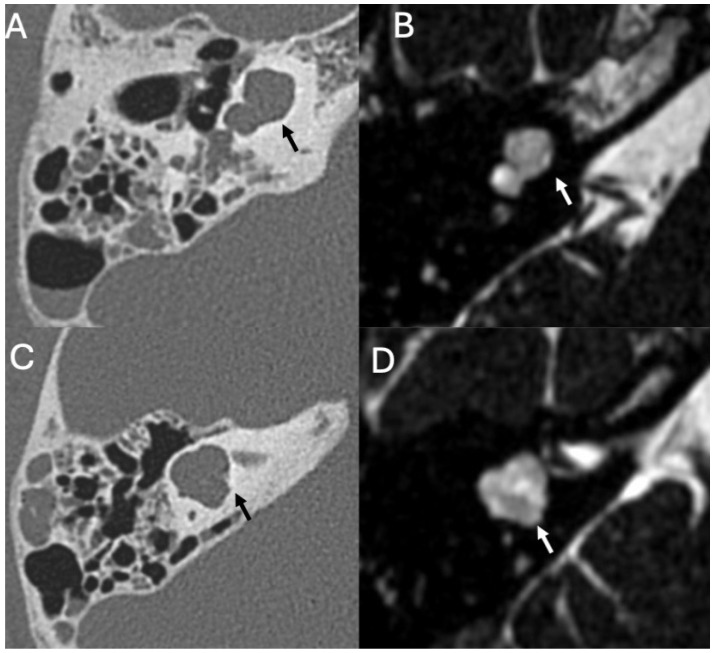
Incomplete partition type I in a 14-year-old patient with recurrent Meningitis due to *Streptococcus pneumoniae*. Axial CT (**A**,**C**) and heavily T2-weighted (**B**,**D**) images show an enlarged cochlea lacking modiolus and interscalar septa, resembling an empty cyst (arrows (**A**,**B**)). The vestibule is enlarged and dysmorphic (arrows (**C**,**D**)). Note also fluid in the mastoid (**A**,**C**).

To reduce the risk of developing Meningitis, it is important to identify the specific type of malformation. Recently, this has been possible thanks to the widespread use of CT and MRI and newborn hearing screening. In the most severe cases, surgical repair should be performed [13]. Among the different types of congenital malformations associated with Meningitis, those developing early during fetal life (6th and 8th weeks of gestation), such as cochlear aplasia, cochlear dysplasia, and common cavity deformity, can predispose to severe recurrent Meningitis due to the formation of a fistula between the subarachnoid space and the middle ear, especially in children [10]. Incomplete partition type I occurs due to an arrest in development during the 5th week of gestation, and is characterized by a cystic cochlea that lacks the entire modiolus and interscalar septum, often accompanied by an enlarged vestibule (Figure 3) [16].

Cochlear hypoplasia type II is characterized by a hypoplastic cochlea with a defective modiolus and interscalar septum. This malformation is associated with abnormal development of the endosteal layer, leading to defects of the stapes footplate and a higher risk of recurrent Meningitis [14,15]. Malformations that appear later in fetal development, such as Incomplete Partition type II, have a partially developed cochlear structure and show a lower risk of developing Meningitis [10,46].

Thin-section high-resolution CT is extremely important to diagnose these malformations and should always be performed in children with Meningitis and deafness [10]. In case of suspected fistula, it is important to evaluate the basal curvature of the cochlea. If it is wider than normal or replaced by an undeveloped sac, the risk of fistula is very high [47]. If there is CSF leakage, surgery should be considered [16].

## 3. Infectious Disease

### 3.1. Otomastoiditis

Otomastoiditis is defined as the inflammation of the middle ear and mastoid, and can be acute or chronic, the latter lasting more than 12 weeks [48]. The middle ear and mastoid are extensions of the upper respiratory tract; thus, they are prone to bacterial invasion through the eustachian tube, generally by *Streptococcus pneumoniae* and *Haemophilus influenzae* [49].

#### 3.1.1. Imaging

**Figure 4:**
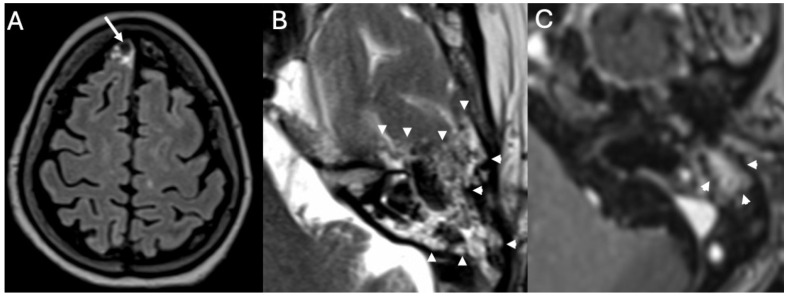
Meningitis due to *Streptococcus pneumoniae* in a 77-year-old patient. Axial enhanced FLAIR (**A**), T2 (**B**), and enhanced T1 (**C**) show leptomeningeal enhancement in the superior frontal region (arrow in (**A**)). There is acute mastoitidis on the left side (arrowheads in (**B**)) with patchy contrast enhancement (arrowheads in (**C**)).

**Figure 5:**
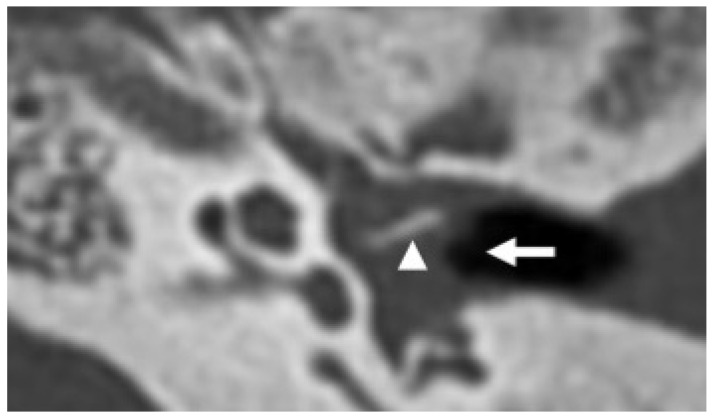
*Varicella-zoster* virus encephalitis in a 20-year-old patient. Axial CT shows fluid in the left middle ear (arrow) due to chronic otitis. There is also marked erosion of the ossicular chain, with the long process of the incus being the only remaining appreciable ear bone left (arrowhead).

**Figure 6:**
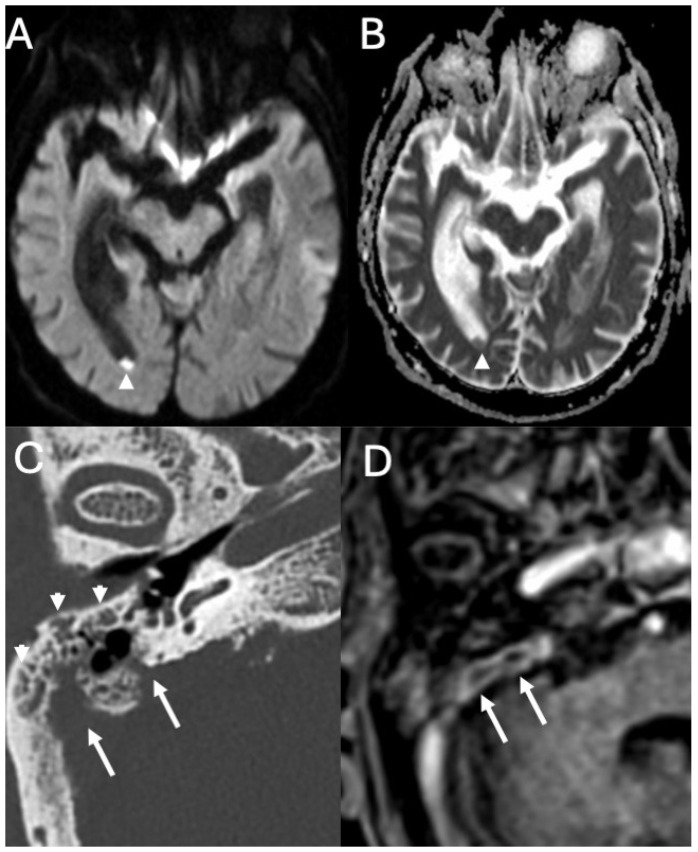
Meningitis due to *Streptococcus pneumoniae* in an 82-year-old. Axial DWI (**A**) with relative ADC map (**B**), CT (**C**), and enhanced T1 (**D**) depict intraventricular purulent material (arrowheads in (**A**,**B**)). Note also right acute mastoiditis (arrowheads in (**C**)) with extensive bony erosion (arrows in (**C**)) and intense contrast-enhancement (arrows in (**D**)).

On CT, acute otomastoiditis appears as opacification of the middle ear and mastoid due to the presence of fluid (Figure 4). On MRI, there is diffuse hyperintensity on T2-weighted and FLAIR sequences of the middle ear and mastoid with mucosal contrast-enhancement and diffusion restriction (Figure 5) [17]. In chronic infection, there are also diffuse bony erosion (Figure 6) and tympanosclerosis, the latter being calcification of the middle ear structures (tympanic membrane, ossicles, ligaments, and muscle tendons) [50]. The tympanic membrane can perforate, whereas the mastoid may become sclerotic [48]. Middle ear granulation tissue, bony erosion, atelectasis, and ossicular chain fixation are also common. High-resolution temporal bone CT is an excellent technique to evaluate the tympanic cavity and the mastoid, and to detect bone erosions, the latter being associated with Meningitis.

#### 3.1.2. Managment

Usually, the antibiotic therapy resolves the infection; otherwise, surgery can be indicated [18]. In about 1–5% of untreated or inadequately treated patients, erosion of the tympanic cavity and mastoid can develop, with a high risk of cerebral complications [7]. Intracranial spread of infection may occur through bony dehiscence, thrombophlebitis, or hematogenous invasion, and may lead to Meningitis, one of the most common complications of otomastoiditis [19]. In the setting of otomastoiditis, Meningitis has been reported to have a prevalence of 35–46.4%, although the studies used to evaluate this data are old and included only 37 people [20]. Rarely, pneumocephalus can be present in otogenic Meningitis [21]. It can be related to cortical defect in the mastoid allowing the passage of air or dissemination by mixed aerobic-anaerobic infective bacteria producing gas [51].

### 3.2. Bony Dehiscence

**Figure 7:**
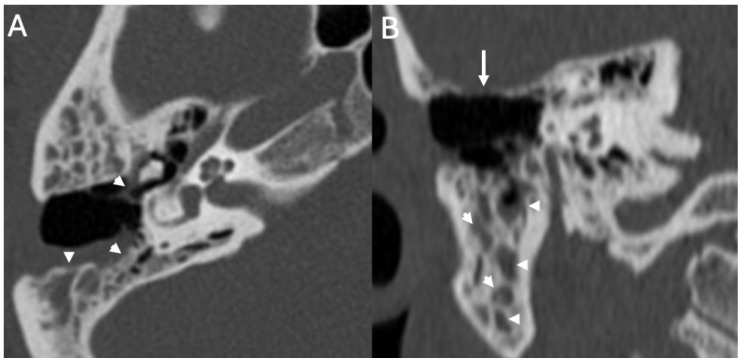
Menigitis due to *Streptococcus pneumoniae* in a 33-year-old. Axial (**A**) and coronal CT (**B**) showing right otomastoid (arrowheads in (**A**,**B**)) and erosion of the tegmen tympani (arrow in (**B**)) The patient also underwent an open tympanoplasty due to cholesteatoma 10 years beforehand.

**Figure 8:**
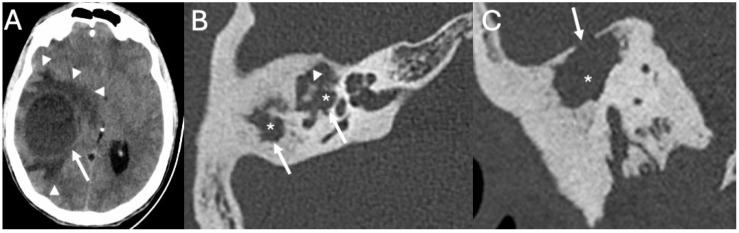
Brain access in a patient with Pneumococcal Meningitis. Axial (**A**,**B**) and coronal CT (**C**) show a brain abscess in the right temporal lobe (arrow in (**A**)) surrounded by extensive amount of vasogenic edema (arrowheads in (**A**)). It was surgically removed right after the exam due to rapid deterioration of the neurological status. It was caused by chronic otomastoiditis on the right side, (arrows in (**B**)) with fluid retention (asterisks in (**B**,**C**)), with extensive erosion of the ossicular chain (arrowhead in (**B**)), and large defect of the tegmen tympani (arrow in (**C**)). Note also diffuse sclerosis of the mastoid due to chronic osteitis.

Bony dehiscence consists of communication between the middle ear and the middle cranial fossa, exposing the subarachnoid spaces to pathological agents from the external environment (Figure 7) [22]. Bony dehiscence can be idiopathic, traumatic, iatrogenic, congenital, or secondary to cholesteatoma or chronic otitis media (Figure 8). Spontaneous dehiscence can affect both the tegmen tympani and the superior semicircular canal (incidence of 36.4%), due to their common embryology [22]. In otogenic Meningitis, the incidence of bony defect is about 73.1%, whereas the incidence of Meningitis as the first manifestation of spontaneous tegmen defects is reported to be 27.3% [20,23]. Temporal bone defects can create a pathway through which meninges and/or cerebral tissue may herniate, resulting in meningocele or meningo-encephalocele. Lim et al. described the association between tegmen tympani defect and cephaloceles with Meningitis and also anecdotally reported very few cases of patients with the triad of otogenic Meningitis, superior semicircular canal dehiscence, and cephalocele through tegmen tympanic defects [24]. High resolution thin-slice CT scan reformatted on the coronal plane is preferred over MRI to investigate bony dehiscence [25].

### 3.3. Cholesteatoma

**Figure 9:**
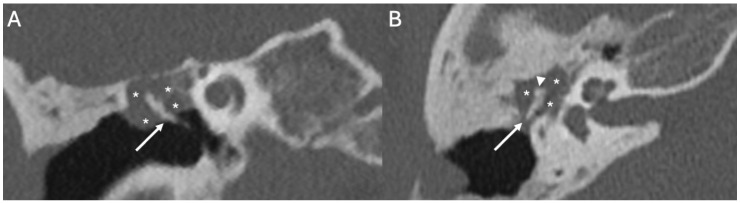
Menigitis due to *Streptococcus pneumoniae* in a 19-year-old patient with recurrent acquired Cholesteatoma. Coronal reformat (**A**) and axial CT (**B**) show hypodense material in the middle ear (epitympanum and mesotympanum, asterisks in (**A**,**B**)), with erosion of the incus (arrows in (**A**,**B**)), and malleus (arrowhead in (**B**)).

Cholesteatoma is a well-defined, non-neoplastic disease resulting from the abnormal proliferation of keratinizing stratified squamous epithelium within the temporal bone [26]. It is usually unilateral, shows a locally aggressive behavior, and can cause erosion of the surrounding structures [52]. Cholesteatoma can be congenital or acquired. The congenital form, rather rare (about 2% of cases), derives from the failure of regression of embryonic epithelial residues. It can be in different regions of the temporal bone, including the middle ear, mastoid, petrous apex, temporal squama, tympanic membrane, and external auditory canal [27]. It typically presents as an epithelial mass, located medially to an intact tympanic membrane. Although congenital, diagnosis often occurs in childhood in the absence of clinical signs such as otorrhea, tympanic perforation, or previous ear surgeries [52]. The acquired form exclusively involves the middle ear and can be primary or secondary. The first one represents most of the cases (about 80%), and it is associated with an apparently intact tympanic membrane, usually being adjacent to the pars flaccida of the tympanic membrane (Prussak recess). Here, a retraction pocket may form that favors the accumulation of keratin derived from epithelial desquamation (Figure 9) [27]. The secondary form (about 20%) develops following the migration of epithelial tissue through a perforation of the tympanic membrane (usually in the pars tensa) secondary to chronic infections, trauma, or previous surgeries [27].

#### 3.3.1. Imaging

**Figure 10:**
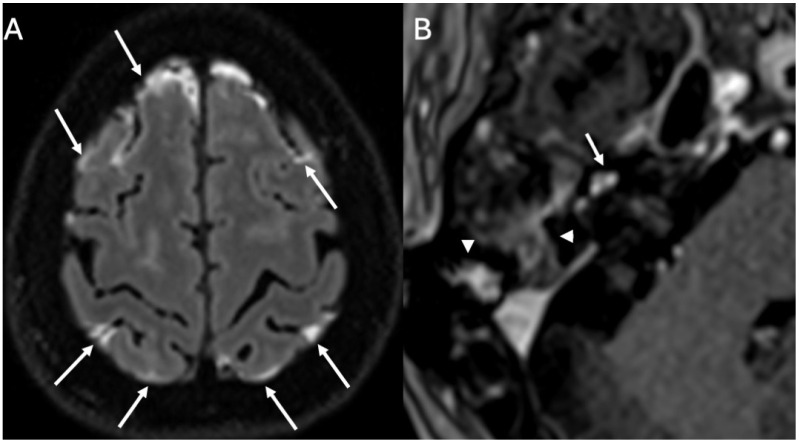
Acute Mastoiditis and labyrintitis in a 64-year-old patient with Hemophilus Influenzae Meningitis. Axial enhanced FLAIR (**A**) and T1 (**B**) show diffuse leptomeningeal enhancement (arrows in (**A**)), enhancement of the right mastoid (arrowheads in (**B**)) consistent with mastoiditis and enhancement of the right cochlea (arrow in (**B**)) due to labyrinthitis.

The first-choice diagnostic investigation in suspected cholesteatoma is high-resolution CT with a bone reconstruction algorithm, in which the lesion appears isodense or slightly hypodense and shows no contrast enhancement [53]. It causes retraction of the tympanic membrane, scutum blunting, and bony erosion, in particular of the tegmen tympani and ossicular chain (Figure 10). Although CT has high spatial resolution and sensitivity, it shows low specificity in the differential diagnosis of cholesteatoma from granulation tissue, secretion, cholesterol granuloma, or neoplasm.

MRI is a complementary investigation to CT, with the cholesteatoma appearing hypointense on T1 and hyperintense on T2 images, without contrast enhancement, but with diffusion restriction, especially on non-echo-planar (EPI) DWI sequences [54]. The latter finding differentiates it from cholesterol granulomas, arachnoid cysts, and mucoceles. Non-EPI DWI lowers magnetic susceptibility distortion and allows better definition. The integration of non-EPI DWI and high-resolution CT is the most effective diagnostic approach, allowing accurate evaluation of the pathology and better surgical planning [54].

#### 3.3.2. Complications

Cholesteatoma can erode bony structures and compromise their integrity, causing conductive hearing loss [55]. Lesion expansion can lead to severe complications, including sigmoid sinus thrombosis, mastoiditis, facial nerve paralysis, perilabyrinthine fistula, cerebrospinal fluid leakage, Meningitis, and brain abscesses [56]. Meningitis is associated with superinfection in acquired forms, and the most common pathogens involved include *Proteus mirabilis*, anaerobic bacteria, *Staphylococcus aureus*, and *Pseudomonas aeruginosa* [30,57]. In a retrospective study, Mustafa et al. reported 12.4% of cases of chronic cholesteatomatous otitis media complicated into Meningitis, although this study has multiple limitations (very inhomogeneous population, single-center and regional, and does not use standardized imaging protocol) [28]. Although the most common form is otogenic Meningitis secondary to infected chronic otitis media, cases of aseptic Meningitis associated with congenital petrous apex cholesteatoma have also been described, as reported by Reardon et al., where rupture of the cholesteatomatous cyst led to the release of keratin and lipid material into the subarachnoid space, resulting in meningeal irritation and recurrent Meningitis [54]. Dubey et al. reported that in a group of 32 patients with intracranial complications from otitis media, 96.8% had cholesteatoma, and in 31.2% of cases, Meningitis occurred [29]. Sun et al. observed that 76.4% of patients with chronic otitis media had cholesteatoma, and in 29.4% of cases, Meningitis developed [30]. Vashishith et al. documented that among 20 patients with intracranial complications, 7 out of 10 showed erosion of the tympanic roof or bone near the sigmoid sinus, with one case of pyogenic Meningitis [58]. All the abovementioned studies have the limit of low number of a patients enrolled; thus, more studies are required to confirm these data.

The pediatric population is considered more vulnerable to developing cholesteatomas and related complications. This is linked to multiple factors, such as immaturity of the Eustachian tube (being shorter, horizontal, and more prone to dysfunction), thinner mastoid and ear ossicles, less effective control of chronic infection and inflammation, high rate of otitis media, and aspecific symptoms [31,59]. Lee et al. reported a prevalence of 0.6% of Meningitis as a complication in children with cholesteatoma, with significantly higher rates in subjects with low socioeconomic status [31].

### 3.4. Labyrinthitis and Vestibulocochlear Neuritis

Labyrinthitis is an inflammation of the membranous labyrinth of the inner ear. The most common causes are infections (such as from otitis media), inflammatory or autoimmune disease, trauma, hemorrhages, and tumors. Labyrinthitis is classified as viral, bacterial (or suppurative), autoimmune, and serous (also known as toxic). In cases of infection, labyrinthitis is most often caused by viral agents such as herpes, measles, mumps, and rubella [32]. In cases of bacterial infection, the most frequent pathogens are *Streptococcus pneumoniae* and *Haemophilus influenzae*. Infections can reach the membranous labyrinth via the bloodstream, through the meninges, or directly from the ear [33]. The symptoms include hearing loss, vertigo, and vomiting, which worsen with sudden movements [60].

Vestibulo–cochlear neuritis is closely related to labyrinthitis, and it is caused by viral infection of one or both vestibular nerves [60]. The incidence in the population is 3.5–15/100,000, and its distribution is equal between both sexes. There is evidence of a correlation between vestibulo–cochlear neuritis and reactivation of *Herpes simplex virus type 1*. Clinically, it is characterized by vertigo, nausea, vomiting, and postural instability [35].

Regarding Meningitis, it can directly cause inflammation of the vestibulocochlear nerves and internal ear structures in almost 30% of patients [1]. It is more common in Meningitis due to *Streptococcus pneumoniae*. It is usually bilateral and may cause permanent deafness. Imaging characteristics of labyrinthitis and neuritis in Meningitis are not specific.

#### Imaging

**Figure 11:**
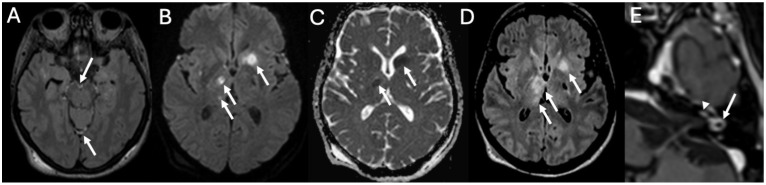
Inner ear involvement in a 64-year-old patient with Pneumococcal Meningitis. Axial FLAIR (**A**), DWI (**B**), ADC (**C**), enhanced FLAIR (**D**), and T1 (**E**) show leptomeningeal enhancement (arrows in (**A**)), multiple ischemic areas (arrows in (**B**–**D**)) and enhancement of the basal turn of the right cochlea (arrowhead in (**E**)) and ipsilateral lateral semicircular canal (arrow in (**E**)) due to labyrinthitis.

MRI is the imaging modality of choice for labyrinthitis, with imaging characteristics varying upon time [6]. In the acute phase, there is contrast enhancement of the structures of the inner ear and acoustic nerves (Figure 10 and Figure 11), whereas in the intermediate phase, there is gradual loss of the physiologic fluid signal on T2-weighted sequences while CT may still appear normal. Finally, if the labyrinthitis does not resolve, it can progress to the ossifying form, with complete loss of the physiologic fluid signal of the inner ear structures due to ossification [34]. The ossification phase may begin in case of chronic inflammation, due to the formation of granulation tissue that may evolve into dense connective tissue obliterating the fluid spaces. Eventually, fibroblasts may differentiate into osteoblast-like cells with ossification of the labyrinthine spaces beginning from the basal turn of the cochlea.

### 3.5. Cochlear Hemorrhage

**Figure 12:**
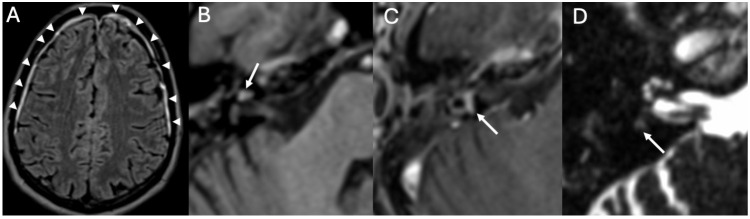
A case of inner ear hemorrhage in a 32-year-old patient with Pneumococcal Meningitis. Axial enhanced FLAIR (**A**), unenhanced T1 (**B**), enhanced T1 (**C**), and heavily T2-weighted sequences (**D**) show diffuse pachymeningeal enhancement (arrowheads in (**A**)), spontaneous T1 hyperintensity of the right cochlea (arrow in (**B**)) caused by hemorrhage and contrast-enhancement of ipsilateral lateral semicircular canal (arrow in (**C**)), which has become fibrotic after two months (arrow in (**D**)). Image readapted from [36].

We described a case of cochlear hemorrhage that occurred in a patient with pneumococcal Meningitis (Figure 12). Cochlear hemorrhage is very rare and usually linked to coagulopathy, which was absent in our patient [36]. As far as we know, this is the first case of cochlear hemorrhage developed as complication of Meningitis. It could be related to vascular damage of small vessels, histopathologically found in patients with pneumococcal Meningitis [37].

## 4. Iatrogenic Causes

In rare cases, ear surgery can increase the risk of developing Meningitis. For instance, it has been reported as rare complication of cochlear implantation. Gowrishankar et al. performed a systematic review to evaluate the risk of developing postoperative Meningitis in this setting [38]. The rate was 0.07%, but the risk was significantly lowered if pneumococcal vaccine and antibiotic prophylaxis were performed. In children (especially those aged less than 5 years), the risk of developing Meningitis after cochlear implantation is 30 times higher than in the general population, and it is even higher in patients with an implant with a positioner or in cases of inner ear malformation with CSF leak [39]. This study has some limits, such as exclusion of non-English papers, the inhomogeneities of the included articles, and the lack of subgroup analysis. Other possible risk factors are otitis media and previous pre-implantation [61]. It usually occurs between 1 and 72 months after surgery [62].

Howitz et al. evaluated the risk of developing bacterial Meningitis following ear, nose, and throat surgery, and reported a higher risk in patients who underwent mastoidectomy, excision of a cholesteatoma, tympanoplasty, and cochlear implantation [40]. In this setting, Meningitis may occur if there is a fistula between the middle ear and the subarachnoid space, reported as possible complication of mastoidectomy [63].

Few case reports have also described Meningitis as an early complication of stapedotomy, developing before the 5th week after surgery [41,64]. Since it has been described in just two case reports, more evidence is required to understand if stapedotomy is linked to higher risk of Meningitis.

## 5. Discussion

**Figure 13:**
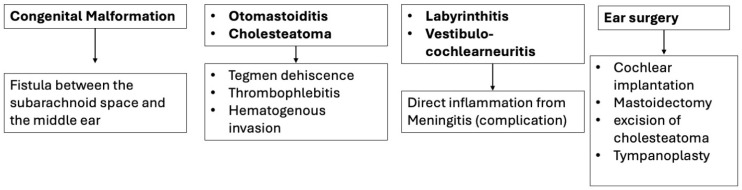
Pathways from ear pathology to Meningitis.

Neuroimaging plays a central role in Meningitis, in particular in detecting ear abnormalities, identifying complications, and guiding therapeutic interventions. Importantly, it integrates radiologic-pathologic correlations that reinforce the need for timely recognition of subtle ear abnormalities, which may otherwise be overlooked during acute Meningitis workup (Figure 13). For instance, recognition of tegmen tympani defects or translabyrinthine fistulas not only explains recurrent Meningitis but also directs preventive surgical repair. Similarly, the documentation of imaging features of cholesteatoma and otomastoiditis strengthens the case for early multidisciplinary management to reduce the high complication burden, including Meningitis and brain abscesses. Therefore, ear-related pathology should not be considered incidental but as a critical factor in Meningitis risk, prognosis, and management. Consequently, children and adults with Meningitis and concurrent hearing loss, CSF leakage, or recurrent infections should undergo high-resolution CT and MRI of the temporal bone to rule out congenital malformations or bony dehiscence, whereas patients with chronic otomastoiditis or cholesteatoma require prompt imaging to detect erosions or intracranial extension before irreversible complications occur.

**Figure 14:**
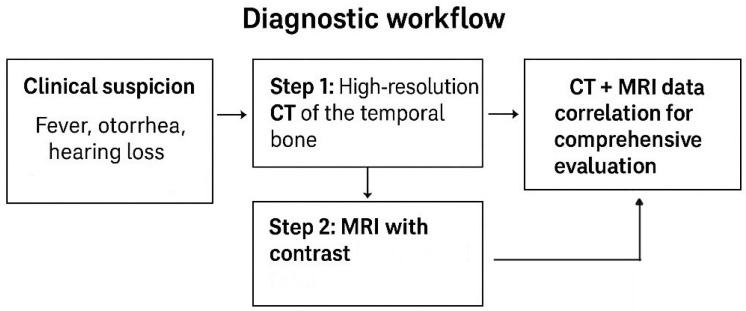
Diagnostic workflow.

Regarding the diagnostic imaging pathway, CT remains the first-line for bony assessment (tegmen tympani, ossicular chain, mastoid involvement). MRI with contrast and non-EPI DWI is essential for identifying soft tissue lesions, inflammatory spread, labyrinthitis, or abscesses. Integration of both modalities provides the highest diagnostic accuracy and guides surgical planning (Figure 14). Early recognition of malformations or fistulas supports preventive surgical intervention, particularly in pediatric patients at high risk of recurrent Meningitis. Otogenic Meningitis should be treated aggressively with antibiotics, but surgery is indicated when imaging shows erosive disease or persistent infection. Post-cochlear implantation patients require strict vaccination protocols and surveillance for Meningitis, especially in younger children or those with malformations. Optimal care requires a multidisciplinary approach with input from radiologists, otolaryngologists, neurosurgeons, and infectious disease specialists.

Another diagnostic challenge is the differentiation of cholesteatoma from chronic inflammatory changes or granulation tissue. CT has high sensitivity for detecting soft-tissue masses and bony erosion but lacks specificity. MRI with non-EPI DWI significantly improves specificity by identifying restricted diffusion typical of cholesteatoma. Still, small lesions (<3 mm) or atypical signal characteristics can lead to false negatives or ambiguous interpretations. This creates ongoing debate about the optimal imaging algorithm, especially in recurrent or residual disease where surgical decision-making hinges on accurate radiologic differentiation. Finally, there is debate on how imaging should weigh against clinical factors. For example, not all temporal bone defects or small cholesteatomas necessitate immediate surgery, yet the risk of Meningitis makes conservative observation difficult to justify. Similarly, radiologic evidence of labyrinthitis may not always correlate with clinical severity, raising uncertainty in prognostication and treatment intensity. Together, these controversies highlight the need for continued refinement of imaging protocols, standardized reporting, and multidisciplinary discussion to optimize care in patients with otogenic Meningitis. When evaluating patients with Meningitis, awareness of possible ear-related causes and complications is essential. For instance, recurrent Meningitis, especially in children, should raise suspicion of congenital ear malformations or CSF leaks. Acute Meningitis with ear symptoms (otorrhea, chronic otitis, hearing loss) suggests otomastoiditis or cholesteatoma. A history of ear surgery or cochlear implant requires consideration of post-surgical fistula or implant-related risk. Despite advances in antimicrobial therapy and imaging, otogenic Meningitis remains a diagnostic and management challenge. One ongoing controversy concerns the extent and timing of imaging evaluation. While high-resolution CT and MRI of the temporal bone are critical for identifying potential intracranial and extracranial complications, there is debate over whether all patients with bacterial Meningitis should undergo routine temporal bone imaging to exclude an otogenic source. Proponents argue that early imaging can identify occult mastoiditis, tegmen defects, or labyrinthine fistulae that may necessitate surgical intervention. Critics, however, highlight the low diagnostic yield in non-otologic Meningitis and the potential for increased healthcare costs and radiation exposure, suggesting that imaging be reserved for patients with otologic symptoms or recurrent infection.

Regarding MRI field strength, 1.5T MRI remains the most practical and reliable one in routine clinical use for otogenic Meningitis. It provides stable, artifact-minimized visualization of the temporal bone, meninges, and labyrinth. 3T MRI can offer higher diagnostic sensitivity if carefully optimized (with spin-echo sequences, localized shimming, and high bandwidth), but it carries a higher risk of susceptibility artifacts in this region. 7T MRI, though promising for neuroanatomical research, is not suitable for routine infection imaging due to excessive inhomogeneity at the skull base. The diagnosis of ear diseases could also be enhanced by the use of artificial intelligence. For instance, it proved useful in cholesteatoma, as such tools showed high sensitivity and specificity for automatic detection of this disease using CT [42].

The literature on otogenic Meningitis is limited by its reliance on small, retrospective case series, often with heterogeneous patient populations and variable diagnostic criteria. Few studies incorporate standardized imaging protocols or correlate imaging findings with microbiological or surgical outcomes. Consequently, the true prevalence of otogenic sources in community-acquired Meningitis may be underestimated, and optimal imaging pathways remain undefined. Moreover, there is limited data on the prognostic value of specific imaging features—such as labyrinthine enhancement, bone erosion, or dural thickening—in predicting complications like abscess formation, venous sinus thrombosis, or persistent neurological deficits. Future research should focus on prospective, multicenter studies using standardized diagnostic algorithms that integrate clinical, microbiological, and imaging data. Development of quantitative imaging biomarkers predictive of meningitic complications (such as diffusion) could refine risk stratification and guide early intervention. In parallel, cost-effectiveness analyses are warranted to evaluate routine versus selective temporal bone imaging in suspected Meningitis, particularly in settings with differing resource availability. Advances in molecular diagnostics and high-resolution imaging may also help delineate the pathogen–host–anatomy interplay underlying otogenic dissemination. Ultimately, a more evidence-based and individualized approach to imaging and surgical decision-making will be essential for improving outcomes in otogenic Meningitis.

The present review is based primarily on narrative synthesis of case reports, retrospective series, and small observational studies, reflecting the limited availability of high-level evidence on otogenic Meningitis. Most of the included publications are single-center and heterogeneous in design, with variable inclusion criteria, diagnostic definitions, and imaging protocols. Consequently, the strength of the conclusions is constrained by the predominance of descriptive data and the absence of prospective, controlled investigations. Few studies provide standardized correlations between imaging findings, microbiological confirmation, and surgical outcomes, which restricts the generalizability of imaging-based recommendations. Furthermore, publication bias toward complicated or surgically treated cases may overestimate the prevalence of specific etiologies or imaging features. Despite these limitations, the aggregated evidence offers valuable insights into radiologic patterns and clinical associations that can guide hypothesis generation and inform future research. Prospective, multicenter studies using standardized imaging criteria and integrated clinical-pathologic validation are needed to establish robust evidence-based guidelines for the diagnosis and management of otogenic Meningitis.

## 6. Conclusions

The ear is frequently implicated in Meningitis, both as a source and as a site of complications. Neuroimaging plays a crucial role in early detection, etiologic assessment, and management planning. High-resolution temporal bone CT or MRI should be performed in Meningitis patients with otologic symptoms (hearing loss, otorrhea, vertigo, mastoid tenderness) or in those with recurrent or unexplained episodes, particularly in children. CT is preferred for evaluating bony integrity, such as tegmen tympani defects, ossicular erosion, or cholesteatoma; MRI is indicated for assessing labyrinthitis, nerve involvement, and intracranial spread. When available, contrast-enhanced MRI with non-EPI DWI offers optimal characterization of soft-tissue lesions and complications. Key imaging red flags for otogenic Meningitis include bony dehiscence of the tegmen tympani or semicircular canal, otomastoid opacification with ossicular or mastoid erosion, restricted diffusion suggesting cholesteatoma or abscess, labyrinthine, or cochlear enhancement indicating labyrinthitis and meningeal enhancement adjacent to temporal bone infection. These findings should prompt multidisciplinary evaluation. Surgical management is indicated when imaging reveals bone destruction, CSF fistula, cholesteatoma, or abscess; medical therapy is appropriate when infection is confined to soft tissues without intracranial extension. In congenital malformations or postoperative leaks, early imaging supports preventive repair to avoid recurrence.

In conclusion, neuroradiologists should routinely evaluate the temporal bones in Meningitis with otologic or recurrent presentation, integrate CT and MRI findings to differentiate otogenic from non-otogenic sources, emphasize red-flag features that warrant urgent ENT or neurosurgical consultation, and adopt structured, recommendation-oriented reporting to guide surgical versus medical management.

## Figures and Tables

**Table 1:** Summary table of key studies. MRI: magnetic resonance imaging; HRCT: high resolution CT; CSF: cerebrospinal fluid.

Topic/Section	Main Reference(s)	Study Design/Type	Key Findings/Contribution
Congenital Inner Ear Malformations	Kimitsuki et al., 1999 [10]; Wani et al., 2012 [11]; Zwierz et al., 2021 [12]; Kivekäs et al., 2015 [13]; Sennaroglu 2010 [14]; Sennaroğlu & Bajin 2017 [15]; Hamano et al., 2018 [16]	Case reports, case series, and reviews	Recurrent meningitis often results from CSF fistulas associated with cochlear dysplasia or incomplete partition anomalies; CT/MRI key for diagnosis and surgical planning.
Otomastoiditis and Otogenic Meningitis	Saat et al., 2015 [17]; Rubini et al., 2024 [18]; Vazquez et al., 2003 [19]; Bruschini et al., 2017 [20]; Barry et al., 2019 [21]	Retrospective studies, imaging reviews, and case reports	HRCT and MRI identify middle ear/mastoid infection and intracranial spread; meningitis occurs in up to 35–46% of untreated cases.
Bony Dehiscence/Temporal Bone Defects	Barbara et al., 2022 [22]; Sanna et al., 2009 [23]; Lim et al., 2012 [24]; Rabiei et al., 2025 [25]	Case-based studies and reviews	Tegmen tympani or semicircular canal defects create a route for meningitis and meningoencephalocele; HRCT coronal reconstructions preferred.
Cholesteatoma and Otogenic Complications	Kuo et al., 2015 [26]; Baráth et al., 2011 [27]; Mustafa et al., 2014 [28]; Dubey et al., 2010 [29]; Sun et al., 2014 [30]; Lee et al., 2020 [31]	Reviews and retrospective studies	Cholesteatoma causes erosion and CSF leak, leading to meningitis (12–30% incidence); MRI improves detection; pediatric risk emphasized.
Labyrinthitis/Vestibulocochlear Neuritis	Taxak & Ram 2020 [32]; Kharrat et al., 2024 [33]; Singh et al., 2023 [34]; Kim et al., 2024 [35]	Case reports and small series	Meningitis can cause secondary labyrinthitis with cochlear enhancement on MRI; progression to ossification possible if chronic.
Cochlear Hemorrhage (Rare Complication)	Perillo et al., 2024 [36]; Engelen-Lee et al., 2016 [37]	Case report; pathologic correlation study	First documented case of cochlear hemorrhage secondary to pneumococcal meningitis; likely due to microvascular injury.
Iatrogenic Causes (Ear Surgery, Cochlear Implants)	Gowrishankar et al., 2023 [38]; Reefhuis et al., 2003 [39]; Howitz & Homøe 2014 [40]; Nielsen & Thomsen 2000 [41]	Systematic review, retrospective cohort, case reports	Post-cochlear-implant meningitis risk = 0.07%; increased in children and malformations; mastoidectomy and stapedotomy occasionally associated.
Artificial Intelligence in Otology	Emilio et al., 2025 [42]	Diagnostic AI validation study	Deep learning on CT shows high accuracy for automatic cholesteatoma detection, supporting imaging standardization.

## Data Availability

Data are available upon reasonable request.

## Abbreviations

The following abbreviations are used in this manuscript:

CSF	Cerebrospinal fluid
CT	Computed tomography
MRI	Magnetic resonance imaging
FLAIR	Fluid-attenuated inversion recovery
EPI DWI	Diffusion restriction on non-echo-planar
